# Assessing the safety and activity of cabozantinib combined with lanreotide in gastroenteropancreatic and thoracic neuroendocrine tumors: rationale and protocol of the phase II LOLA trial

**DOI:** 10.1186/s12885-023-11287-2

**Published:** 2023-09-26

**Authors:** Francesca Corti, Maria Pia Brizzi, Vito Amoroso, Dario Giuffrida, Francesco Panzuto, Davide Campana, Natalie Prinzi, Massimo Milione, Tommaso Cascella, Carlo Spreafico, Giovanni Randon, Simone Oldani, Rita Leporati, Giulia Scotto, Iolanda Pulice, Benedetta Lombardi Stocchetti, Luca Porcu, Jorgelina Coppa, Maria Di Bartolomeo, Filippo de Braud, Sara Pusceddu

**Affiliations:** 1 Department of Medical Oncology, Fondazione IRCCS Istituto Nazionale Dei Tumori Di Milano, ENETS Center of Excellence, Via Giacomo Venezian 1, 20133 Milan, Italy; 2 Azienda Ospedaliera Universitaria San Luigi Gonzaga, Orbassano, Italy; 3 Medical Oncology Unit, Department of Medical & Surgical Specialties, Radiological Sciences & Public Health, University of Brescia at Spedali Civili Hospital, Brescia, Italy; 4 Medical Oncology Department, Istituto Oncologico del Mediterraneo, Catania, Viagrande Italy; 5 Department of Medical-Surgical Sciences and Translational Medicine, Sapienza University of Rome, Digestive Disease Unit, ENETS Center of Excellence, Sant’ Andrea University Hospital, Rome, Italy; 6 Division of Medical Oncology, IRCCS Azienda Ospedaliera- Universitaria Bologna, NET Team Bologna, ENETS Center of Excellence, Bologna, Italy; 7 First Division of Pathology, Department of Pathology and Laboratory Medicine, Fondazione IRCCS Istituto Nazionale Dei Tumori Di Milano, Milan, Italy; 8 Department of Radiology, Fondazione IRCCS Istituto Nazionale Dei Tumori Di Milano, ENETS Center of Excellence, Milan, Italy; 9 Clinical Trial Center, Fondazione IRCCS Istituto Nazionale Dei Tumori Di Milano, Milan, Italy; 10 Methodology for Clinical Research Laboratory, Oncology Department, Istituto Di Ricerche Farmacologiche Mario Negri IRCCS, Milan, Italy; 11 Gastro-Entero-Pancreatic Surgical and Liver Transplantation Unit, Fondazione IRCCS Istituto Nazionale Dei Tumori Di Milano, ENETS Center of Excellence, Milan, Italy; 12 Department of Oncology and Hemato-Oncology, Università Degli Studi Di Milano, Milan, Italy

**Keywords:** Neuroendocrine tumors, Lanreotide, Cabozantinib, Somatostatin analogs, Tyrosine kinase inhibitors, Clinical trial

## Abstract

**Background:**

Well-differentiated (WD) neuroendocrine tumors (NETs) are a group of rare neoplasms with limited therapeutic options. Cabozantinib is an inhibitor of multiple tyrosine kinases with a pivotal role in NET pathogenesis, including c-MET and Vascular Endothelial Growth Factor Receptor 2 (VEGFR2). LOLA is the first prospective phase II trial aiming to assess the safety and activity of cabozantinib combined with lanreotide in WD NETs of gastroenteropancreatic (GEP), thoracic and of unknown origin.

**Methods:**

This is a multicenter, open-label, double-cohort, non comparative, non-randomized, three-stage phase II trial. Eligible patients have to meet the following inclusion criteria: diagnosis of advanced or metastatic, progressive, non-functioning WD thoracic NETs, GEP-NETs or NETs of unknown origin with Ki67 ≥ 10%; positive 68 Ga-PET uptake or somatostatin receptor 2 immunohistochemical (IHC) stain; maximum 1 prior systemic regimen for metastatic disease. Two cohorts will be considered: pNETs and carcinoids (typical or atypical lung and thymus NETs, gastro-intestinal NETs or NETs of unknown origin). In stage I, the primary objective is to find the optimal dose of cabozantinib in combination with lanreotide and to evaluate the safety of the combination (percentage of patients experiencing grade 3–5 toxicities according to NCI-CTCAE version 5.0). Starting dose of cabozantinib is 60 mg/day continuously, plus lanreotide 120 mg every 28 days. In stage II and III, co-primary endpoints are safety and overall response rate (ORR) according to RECIST version 1.1. The uninteresting antitumor activity is fixed in ORR ≤ 5%. Secondary endpoints are progression-free survival and overall survival. Exploratory objectives include the assessment of c-MET, AXL and VEGFR2 IHC expression, to identify predictive or prognostic tissue biomarkers. Enrolment started in July 2020, with an expected trial duration of 42 months comprehensive of accrual, treatment and follow-up. Considering a drop-out rate of 5%, the maximum number of enrolled patients will be 69.

**Discussion:**

Supported by a solid rationale, the trial has the potential to generate milestone data about the synergistic effects of cabozantinib plus lanreotide in a group of NET patients with relatively aggressive disease and limited therapeutic options.

**Trial registration:**

LOLA is registered at ClinicalTrials.gov (NCT04427787) and EudraCT (2019–004506-10).

## Background

Neuroendocrine tumors (NETs) are rare malignant neoplasms that arise from diffuse neuroendocrine cells. As a result of increased detection rate, improved diagnostic accuracy and advances in systemic therapies, the annual age-adjusted incidence of NETs increased from 1.09/100000 persons per year in 1973 to 6.98/100000 in 2012, with a parallel rise in prevalence [1].

Approximately 80 to 90% of well-differentiated (WD) gastroenteropancreatic (GEP) and thoracic NETs express somatostatin receptors (SSTRs) on their cell surface, that bind with high affinity somatostatin analogs (SSAs) lanreotide autogel and octreotide long-acting release (LAR). These drugs are the mainstay for the control of NET-associated endocrine syndromes and represent one pivotal standard treatment option for patients with advanced NETs expressing SSTRs [2–4]. Based on primary site and histopathological features, WD NETs can display heterogeneous clinical behavior and prognosis, grade (G)2 and G3 NETs being more aggressive than the indolent G1 forms [5]. New combinations of SSAs and other investigational drugs are therefore warranted, with the aim to improve clinical outcomes, while maintaining a good tolerability profile.

Combined inhibition of receptor tyrosine kinases and angiogenesis has already proven an effective strategy in the treatment of advanced NETs. The multikinase inhibitors sunitinib and pazopanib—targeting Vascular Endothelial Growth Factor Receptor (VEGFR) 2–3, platelet-derived growth factor receptors (PDGFR α/β), and c-KIT—showed clinical activity in advanced NETs. Sunitinib has been approved for the treatment of advanced pancreatic (p)NETs based on the results of a phase III randomized clinical trial [6–8].

Current research is increasingly focusing on the development of new multitarget tyrosine kinase inhibitors (TKIs), with the aim to improve therapeutic efficacy. Surufatinib (a VEGFR 1–3, fibroblast growth factor receptor (FGFR) -1 and colony-stimulating-factor-1 receptor inhibitor) has recently shown a progression-free survival (PFS) advantage over placebo in two randomized phase III trials in advanced NETs of pancreatic and extra-pancreatic origin [9, 10]. Similarly lenvatinib (a VEGFR 1–3, FGFR1-4, PDGFRα, RET and KIT inhibitor) showed interesting activity in pre-treated WD NET patients in a phase II trial, whereas axitinib (a VEGFR 1–3 inhibitor) combined with octreotide LAR improved response rates and PFS outcomes over placebo in a randomized phase II/III study [11, 12]. More recently, results of the prospective randomized placebo- controlled phase II Alliance A021202 trial of pazopanib (an oral VEGFR-2,-3, PDGFR-α, and β, and c-KIT inhibitor) in patients with progressive carcinoid tumors demonstrated a PFS advantage in the interventional arm over the placebo arm [13].

Cabozantinib is an orally administered small molecule that inhibits the enzymatic activity of multiple tyrosine kinases including c-MET, VEGFR2, RET, KIT, AXL, and Fms Related Tyrosine Kinase 3 [14]. Recent studies suggest that c-MET signaling may play a role in NET biology and pathogenesis. c-MET protein overexpression and increased mRNA levels were detected in 17–33% of primary GEP-NETs and in up to 60% of metastatic sites. c-MET levels correlate with increased Ki-67 proliferation index, increased risk of developing liver metastases and decreased survival in GEP NETs [15–18]. c-MET and/or its phosphorylated form were also described in typical and atypical lung carcinoids, with more than 80% of cases showing a strong expression [19].

**Fig. 1:**
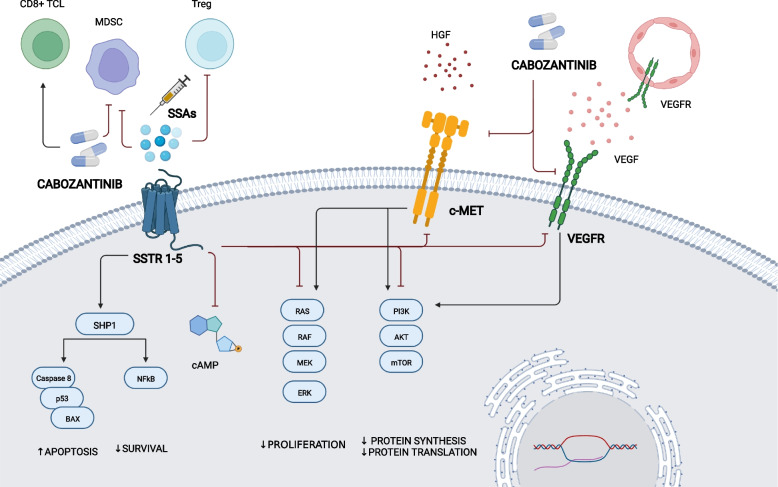
Synergistic antiproliferative, anti-angiogenetic and immune-modulatory effects of cabozantinib combined with somatostatin analogs. cAMP: cyclic adenosine monophosphate; ERK: extracellular signal-regulated kinases; HGF: hepatocyte growth factor; NFkB: nuclear factor kappa-light-chain-enhancer of activated B cells; MDSC: myeloid-derived suppressor cell; mTOR: mammalian target of rapamycin; PI3K: phosphoinositide 3 kinase; RAS: Rat sarcoma virus; RAF: Rapidly Accelerated Fibrosarcoma; SHP1: Src homology region 2 domain-containing phosphatase-1; SSA: somatostatin analog; SSTR: somatostatin receptor; TCL: T cell Lymphocyte; Treg: regulatory T cell; VEGF(R): Vascular Endothelial Growth Factor (Receptor)

The biological rationale of the synergistic effects of SSAs in combination with cabozantinib resides in the concomitant inhibition of intracellular signaling pathways associated with tumor cell proliferation, angiogenesis and immune modulation. The antiproliferative effects of SSAs are mediated by the inhibition of the Ras/mitogen-activated protein kinase (MAPK) and phosphoinositide 3 kinase (PI3K)/Akt/mammalian target of rapamycin (mTOR) cascades. In vitro*,* SSTR2 enhances the dephosphorylation of PI3K and Akt, negatively affecting mTOR-mediated protein synthesis and translation. Moreover, the simultaneous stimulation of SSTR 2 and 5 achieves significant inhibition of Ras and extracellular signal-regulated kinase (ERK)-1/2. [20–23] Similarly, cabozantinib inhibits the activity of VEGFR2, c-MET and AXL at the level of both cancer cells and peritumoral vessels, thus leading to the downstream inhibition of PI3K/Akt/mTOR and Ras/MAPK, with antiproliferative and antiangiogenic effects. [24] Also SSAs exert anti-angiogenic effects by engaging SSTRs on the vascular endothelium and by downregulating the secretion of proangiogenic factors, such as VEGF and PDGF [25]. Immune modulation has been advocated as a part of the antitumor effect of several targeted therapies, including cabozantinib and SSAs. Cabozantinib treatment in combination with SSAs may therefore modulate circulating immune populations and the tumor immune infiltrate, potentially reverting the tumor-associated immunosuppressive phenotype, and strengthening the biological rationale of exploiting this combination in NETs [26–31] **(**Fig. 1).

The safety and the activity of cabozantinib in NETs has been assessed in a recent phase II trial, that evaluated cabozantinib at the dose of 60 mg/day in 61 patients with progressive, WD G1-2 carcinoids (41 patients) and pNETs (20 patients). Overall response rate (ORR), which represented the primary endpoint of the study, was 15% both in the pNET (95% Confidence Interval [CI] 5–36%) and carcinoid groups (ORR 15%, 95% CI: 7–28%). Median (m) PFS was 21.8 months (95% CI: 8.5–32.0) in patients with pNETs and 31.4 months (95% CI: 8.5- not reached) in patients with carcinoids. Although dose reductions were required in 81% of the 53 patients completing more than 1 treatment cycle, treatment proved tolerable. The most frequent G 3/4 toxicities included hypertension (13%), hypophosphatemia (11%) and diarrhea (10%) [14].

Based on these encouraging preliminary data, further evaluation of cabozantinib in advanced NET patients is warranted. Exploiting new combinations of SSAs and targeted agents, such as cabozantinib, with potential synergistic activity represents an issue of particular clinical interest especially in the relatively high-risk population of G2-G3 WD NET and thoracic carcinoids, for whom therapeutic options are limited and SSA monotherapy may be insufficient to guarantee adequate and durable disease control.

The LOLA trial is the first prospective phase II study aiming to assess the safety and activity of cabozantinib in combination with lanreotide in WD G2-G3 NETs of GEP and of unknown origin with Ki-67 ≥ 10% and in thoracic carcinoids, with the aim to evaluate as secondary endpoints the impact of this combination on PFS and overall survival (OS).

Here we report the methodological study protocol of the LOLA trial (NCT04427787).

## Methods

### Study design

This is a multicenter, open-label, double cohort, non comparative, non-randomized, three-stage phase II trial aiming to assess the safety and activity of the combination of cabozantinib and lanreotide in patients with unresectable, advanced or metastatic WD G2-3 GEP-NETs with Ki-67 ≥ 10% (including pancreatic NET, small intestine NET, stomach NET, colon and rectum NET), thoracic NETs (including typical and atypical carcinoids of the lung and thymus) independently of proliferation index and NETs of unknown origin with Ki-67 ≥ 10%.

A list of participating Centers is reported in Table 1.

**Table 1:** Italian recruiting centers of the LOLA trial

N°	Center	Site	Principal Investigator
1	Fondazione IRCCS Istituto Nazionale dei Tumori di Milano	Milan (Italy)	Sara Pusceddu, MD
2	Policlinico Sant'Orsola Malpighi	Bologna (Italy)	Davide Campana, MD
3	Azienda Ospedaliera Universitaria Sant’Andrea	Rome (Italy)	Francesco Panzuto, MD
4	Istituto Oncologico del Mediterraneo -IOM	Catania (Italy)	Dario Giuffrida, MD
5	Azienda Ospedaliera Universitaria San Luigi Gonzaga	Orbassano (Italy)	Maria Pia Brizzi, MD
6	ASST-Spedali Civili	Brescia (Italy)	Vito Amoroso, MD

### Study population

Adult patients (male or female) that are 18 years of age or older will be enrolled according to the following inclusion criteria: diagnosis of unresectable, advanced or metastatic non-functioning WD GEP-NET (including G2-G3 pNET, small intestine NET, stomach NET, colon and rectum NET) with Ki-67 ≥ 10%, WD thoracic NET (typical and atypical carcinoids of the lung and thymus) independently of proliferation index, or WD NET of unknown primary with Ki-67 ≥ 10%; locally advanced or metastatic disease documented as progressive by response evaluation criteria in solid tumors (RECIST) version 1.1. on computed-tomography (CT)-scan or magnetic resonance imaging (MRI) within 12 months prior to baseline [32]; documented Octreoscan/positron emission tomography (PET) Ga68 uptake according to the Krenning score [33] within 6 months before study entry or SSTR2 immunohistochemical stain [34] on pretreatment/archival tumor tissue samples; Eastern Cooperative Oncology Group (ECOG) performance status (PS) 0 or 1; treatment naïve patients or patients who have received maximum 1 prior systemic regimen for metastatic disease (prior peptide receptor radionuclide therapy must be completed at least 6 months prior to enrolment; prior treatment with SSAs, biologic therapy including everolimus or TKIs, immunotherapy, chemotherapy, ablative therapies and/or radiation must be completed at least 28 days prior to registration); adequate liver, kidney, bone marrow and thyroid function.

Patients must not meet any of the following exclusion criteria: poorly differentiated neuroendocrine carcinomas; prior treatment with dose superior or equal to 120 mg per month of lanreotide; prior treatment with cabozantinib; known brain metastases or cranial epidural disease unless adequately treated with radiotherapy and/or surgery, stable for at least 3 months before study entry and neurologically asymptomatic in the absence of corticosteroids; history of cardiac angioplasty or stenting, myocardial infarction, unstable angina, symptomatic peripheral vascular disease, New York Heart Association Class III or IV congestive heart failure, cerebrovascular accidents, active bleeding or bleeding diathesis, pulmonary embolism or untreated deep venous thrombosis within the past 6 months; history of aneurysms and arterial dissections; poorly controlled hypertension.

### Study treatment

Cabozantinib will be administered orally at the starting dose of 60 mg/day continuously, in combination with lanreotide 120 mg administered through deep subcutaneous injection every 28 days. Both treatments will start the same day. When cabozantinib dose reduction is necessary, it is recommended to reduce from 60 mg daily to 40 mg daily, and then to 20 mg daily. The dose of lanreotide will be fix thorough the study (standard dose).

### Study objectives and endpoints

The primary objective of the study is to demonstrate the safety, tolerability and activity of the association of cabozantinib and lanreotide in patients with WD GEP-NETs, thoracic NETs and NETs of unknown primary. Trial design consists of three stages.

In the I (run-in) stage, the primary objective is to evaluate the safety and tolerability of the combination of cabozantinib plus lanreotide, defined as the percentage of patients experiencing G3-5 toxicities according to National Cancer Institute-Common Terminology Criteria for Adverse Events (NCI-CTCAE) version 5.0.

In II and III stage co-primary objectives will be: **a**. activity of the combination of cabozantinib plus lanreotide in terms of ORR, defined as the proportion of patients whose confirmed best overall response is either a partial response or complete response, based on investigator-assessed RECIST criteria version 1.1; b. safety and tolerability of the combination defined as percentage of G3-5 toxicities according to NCI-CTCAE version 5.0.

Secondary objectives (stages II and III) will be to evaluate clinical efficacy of cabozantinib in combination with lanreotide in terms of: a. PFS, defined as the time from first day of treatment administration until disease progression according to RECIST criteria version 1.1 or death from any cause, whichever occurs first; b. OS, defined as the time from first day of treatment administration until death from any cause.

### Translational objectives

Exploratory objectives include the investigation of the prognostic and predictive role of tissue biomarkers. As mandatory inclusion criteria, representative formalin fixed paraffin embedded tissue (either archival or recently biopsied prior to study entry) collected from the primary tumor and/or a metastatic site will be analyzed by immunohistochemistry to determine VEGFR2, MET, AXL expression levels and their association with ORR and time-to-event endpoints (i.e. PFS and OS). In addition, tumor tissue may be used to explore other possible predictors of outcomes in WD NETs receiving lanreotide plus cabozantinib, with the aim to select and identify tissue biomarkers modulating treatment activity and/or safety.

### Sample size and statistical design

**Fig. 2:**
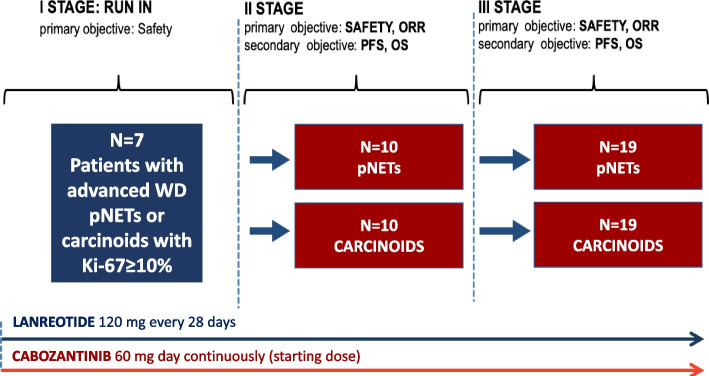
Study design N: number; pNETs: pancreatic neuroendocrine tumors; ORR: Overall response rate; OS: overall survival; PFS: progression-free survival; WD: well-differentiated

The statistical design is adaptive, according to three sequential stages (see the study flow-chart depicted in Fig. 2).

Two cohorts of patients will be enrolled in the study according to different histotypes: the first cohort includes pNETs; the second cohort includes carcinoids (i.e. small intestine NETs, stomach NETs, colon and rectum NETs, typical and atypical lung and thymus NETs) and NETs of unknown origin. Enrolment in the pancreatic and carcinoid arms will be competitive in the first stage (i.e. run-in stage).

*I stage.* The primary objective of I stage is to find the optimal dose of cabozantinib in combination with lanreotide and to evaluate the safety and the tolerability of the combination. Starting dose of cabozantinib is 60 mg daily plus lanreotide 120 mg every 28 days. No more than 7 patients will be enrolled in the I stage. If G3-5 adverse events (AEs)—excluded not clinically relevant laboratory abnormalities according to NCI-CTCAE version 5.0 or clinically manageable and reversible G3 AEs within 7 days of onset—are observed in more than one patient out of the first seven enrolled patients, cabozantinib starting dose of 60 mg will be considered too toxic and ruled out (H_0_: % grade 3–5 toxicities ≤ 5%; nominal p-value: 0.05; actual p-value: 0.044) and 40 mg will be chosen as starting dose of cabozantinib in the subsequent stage. Otherwise, 60 mg will be chosen as starting dose of cabozantinib in the subsequent stage. An interim safety analysis will be performed when 7 patients will be observed on treatment for at least 8 weeks.

In order to preserve internal validity, outcomes’ assessment will be re-started de novo in the II stage (i.e. I and II stages are distinct phases of an operationally seamless, not inferentially seamless design).

*II-III stage.* In stages II-III the optimal Simon two-stage design will be used for both cohorts [35]. The primary endpoint of the II and III stages is ORR according to RECIST version 1.1. Co-primary endpoint will be the evaluation of the percentage of G 3–5 toxicities according to NCI-CTCAE version 5.0. Secondary endpoints will be PFS and OS.

The uninteresting antitumor activity (H_0_) is fixed in ORR ≤ 5%. The useful antitumor activity to be detected (H_1_) is fixed in ORR ≥ 20%. For both arms (pNET and carcinoids), 10 patients will be accrued in the stage II. If no responses are observed in these 10 patients, the arm will be stopped (nominal type II error = 0.20). Otherwise, other 19 patients will be accrued in stage III, for a total of 29 patients. The null hypothesis will be rejected if 4 or more responses are observed in 29 eligible patients (nominal type I error = 0.05). Considering a drop-out rate of 5% (i.e. eligible patients without the assessment of objective response) the maximum number of enrolled patients will be 69 (including the 7 patients included in the interim analysis after stage I).

Regarding the safety co-primary endpoint, the null hypothesis to be rejected is a percentage of AEs greater or equal to 20% (i.e. H_0_: ≥ 20%, nominal type I error = 0.05) against an alternative hypothesis H_1_: ≤ 5%. Because the statistical design is adaptive (i.e. a couple of Simon’s two stage designs are planned after the first run-in stage), the total number of patients that could be analyzed for safety is uncertain prior to study execution. It will be 20 patients if both arms stop at stage II, 39 patients if only one arm stops at stage II and 58 patients if both arms participate to stage III. Thresholds to identify significant level at 5% and exact binomial test’s power will be determined based on the real number of patients to be analyzed (i.e. 0 out of 20 patients [test’s power: 36%], 3 out of 39 patients [test’s power: 87%]; 6 out of 58 patients [test’s power: 97%] are the maximum number of patients with observed G3-G5 AEs to reject H0: ≥ 20% at a 5% significant level). Patients who did not receive treatment for any cause or severely violated protocol inclusion/exclusion criteria will be excluded from the primary and secondary analyses of all stages.

### Study procedures

Baseline assessments, including a 12-lead electrocardiogram (ECG) with QTc interval evaluation, a multigated acquisition (MUGA) scan or cardiac echocardiogram and radiological evaluation (CT or MRI of the chest, abdomen and pelvis) should be obtained within 28 days prior to start of study treatment. All additional suspected sites of disease (brain, bone) should be imaged at baseline.

Disease assessment will be performed every 3 cycles (time window for the scans is ± 28 days) with the same imaging modalities used at baseline. A blinded Central Review of imaging will be performed.

Subjects will be monitored continuously for AEs while on study from the time of signing informed consent through 30 days after the date of the decision to permanently discontinue study treatment.

All patients who are discontinued from study drug for any reason other than disease progression will continue to have tumor assessments as per study schedule until documented disease progression or until the initiation of new anticancer therapy. Table 2 reports study procedures and assessments.

**Table 2:** Study procedures and assessments

	Stage I-II-III				
	**Baseline**-28 day to 0 day	**At each Cycle**	**Every 3 Cycles**	**End of Treatment**	**Follow-up (every 3 months)**
Informed Consent	X				
Demography	X				
Inclusion/Exclusion criteria	X				
Diagnosis	X				
Prior therapy	X				
Medical history	X				
Physical Examination^a^	X	X	X	X	
Vitals signs^a,^^b^	X	X	X	X	
ECOG/Karnofsky Performance Status^a^	X	X	X	X	
Cell blood count, Chemistries, Pregnancy test, Thyroid function test, Coagulation test, Urinalysis^a^	X	X	X	X	
HbA1c, insulin, HDL/LDL-cholesterol, triglycerides, B12-cianocobalamin, D-vitamin, Parathormon, Chromogranin A, NSE	X		X	X	
Hepatitis B core antibody, Hepatitis B surface antigen, and Hepatitis C antibody	X				
HBV DNA or HCV RNA^c^	(X)				
ECG with QTc interval	X		X		
ECHO or MUGA	X				
Archival or recently biopsied tumor tissue	X				
CT scan /MRI^d^	X		X		
Bone scan or Xray^e^	(X)		(X)		
68 Ga-PET or Octreoscan^f^ or IHC for SSTR2	X				
Drug accountability		X	X	X	
Concomitant medications^a^	X	X	X	X	
Adverse Events^a^	X	X	X	X^g^	
Survival data					X

*CT* computed tomography, *ECG* electrocardiogram, *ECHO* echocardiography, *ECOG* Eastern Cooperative Oncology Group, *HBV* Hepatitis B virus, *HCV* Hepatitis C virus, *HDL* high density lipoprotein, *IHC* immunohistochemistry, *LDL* low density lipoprotein, *MRI* magnetic resonance imaging, *MUGA* multigated acquisition, *NSE* neuron specific enolase, *PET* positron emission tomography, *SSTR2* somatostatin receptor 2

^a^To be performed every 2 weeks (Cycle 1 day 1, Cycle 1 day 14, Cycle 2 day 1, Cycle 2 day 14) for the first 8 weeks, and every 4 weeks thereafter (at each cycle)

^b^Weight, height, blood pressure, heart rate, respiratory rate, SpO2, temperature

^c^Only in subjects who have documented HBV or HCV positivity at baseline

^d^Abdominal/thorax radiological assessment with CT or MRI should be performed at baseline and every 3 cycles (with a time window of ± 28 days). CT or MRI of the head must be conducted to rule out brain metastases (only at screening and when clinically indicated). The same radiological imaging modalities are to be used as during study

^e^Not mandatory at baseline, but should be obtained if bone metastases are suspected or documented. If bone metastases at baseline are confirmed, patients will repeat bone scan or specific X-ray every 3 cycles (time window ± 28 days)

^f^Within 6 months before study entry

^g^Adverse events will be reported up to 30 days after the last dose of study treatment. Serious adverse events will be reported through 90 days of discontinuation of study treatment

### Safety

All AEs that occur between the first study-related procedure and 30 days after the last dose of study drug will be reported in the source document and in electronic Case Report Forms. Serious adverse events (SAEs) are defined as any AE that results in death, that is life threatening, that places the subject at immediate risk of death or that results in hospitalization, prolongation of existing hospitalization or in a persistent significant disability. All SAEs that occur following the subject’s written consent to participate in the study through 90 days of discontinuation of dosing must be reported to the Sponsor within 24 h after learning of the event, regardless of relationship to study treatment. Expedited reporting will follow local and international regulations.

Safety data will be reviewed by Data Monitoring Committee on a periodic basis, approximately every 3 months from the date of first-patient-in.

### Ethics and regulatory considerations

The study is conducted in full conformance with the International Conference on Harmonisation (ICH) E6 guideline for Good Clinical Practice (GCP), the principles of the Declaration of Helsinki and according to the laws and regulations of the country in which the research is conducted.

The study has been approved by the Institutional Review Board/Independent Ethics Committee (IRB/IEC) of the Coordinating Center (Fondazione IRCCS Istituto Nazionale dei Tumori di Milano) and by the local Ethics Committees of participating centers. The study has been registered in EudraCT database (2019–004506-10) and at clinicaltrials.gov (NCT04427787). Written informed consent will be obtained as a prerequisite for study entry.

### Recruitment

Enrolment started in July 2020 and is currently ongoing, with an expected trial duration of 42 months comprehensive of accrual, treatment and follow-up.

## Discussion

Cabozantinib is an oral multitarget TKI approved for the treatment of advanced hepatocellular carcinoma (HCC) in patients who have previously been treated with sorafenib, for advanced or metastatic differentiated thyroid carcinoma in patients refractory or not eligible to radioactive iodine who have progressed on prior systemic therapy, and for advanced intermediate or poor risk renal cell carcinoma (RCC)—as first-line treatment or following prior VEGF-targeted therapy [36–39]. In the advanced RCC setting, cabozantinib was shown to improve PFS outcomes compared to everolimus and sunitinib in the pretreated and first-line settings, respectively [36, 37].

Sunitinib is the only multitargeted TKI approved for advanced pNETs [6]. Similarly to sunitinib, cabozantinib inhibits the tyrosine kinase enzymatic activities of multiple receptors including VEGFR2, MET, AXL, and RET, which have been implicated in tumor proliferation, survival and neoangiogenesis [14, 24, 25]. Based on the efficacy and activity data from phase II and III trials in RCC, HCC, thyroid carcinoma and NET patients [14, 36–39], due to its high-spectrum biological activity against multiple and non-redundant oncogenic pathways, cabozantinib may be superior to other angiogenesis inhibitors and provide clinical benefit in the treatment of rare tumors with few available treatment options, such as NETs. Other multitarget TKIs (surufanitib, lenvatinib, pazopanib) have recently shown promising activity and efficacy in patients with metastatic NETs, thus corroborating the investigation of such agents in this setting [9–11, 13]. The activity of single-agent cabozantinib has been investigated in a phase II study in G1-2 carcinoids and pNETs, and this agent is currently being investigated in patients with advanced NETs progressing on prior therapy in the phase III randomized placebo-controlled CABINET trial (NCT03375320). However, to date, no specific data have been published with regard to the relatively high-risk population of G2-G3 WD NET, for whom combinations of SSAs and potentially synergistic targeted agents represent an unmet clinical need.

In our opinion, the combination of cabozantinib with other agents such as SSAs may result in synergistic antiproliferative effects, potentially enhancing antitumor activity and clinical outcomes. The biological rationale of the synergistic effects of cabozantinib in combination with SSAs resides in the concomitant inhibition of intracellular signaling pathways associated with tumor cell proliferation, angiogenesis and immune modulation [15–31].

LOLA is the first multicenter, open-label, phase II trial aiming to prospectively define the safety and activity of a concomitant treatment with cabozantinib and lanreotide in progressive advanced WD thoracic NETs, G2-3 GEP-NETs and NETs of unknown origin with Ki-67 ≥ 10%. Supported by a solid biological and clinical rationale, the trial has the potential to generate milestone data about the possible synergistic effects of this combination in a population of NET patients with relatively aggressive disease, for whom therapeutic options are limited and an optimal treatment sequence is not yet defined. Moreover, biomarkers’ investigation may allow to identify prognostic tools associated with treatment benefit to better inform patient’s selection for this treatment strategy.

## Data Availability

Not applicable.

## Abbreviations

AEs: adverse events
CI: Confidence Interval
CT: computed-tomography
ECG: electrocardiogram
ECOG: Eastern Cooperative Oncology Group
ERK: extracellular signal-regulated kinase
FGFR: fibroblast growth factor receptor
G: grade
GEP: gastroenteropancreatic
HCC: hepatocellular carcinoma
ICH: GCP International Conference on Harmonisation E6 guideline for Good Clinical Practice
IRB/IEC: Institutional Review Board/Independent Ethics Committee
m: Median
MAPK: Mitogen-activated protein kinase
MRI: Magnetic resonance imaging
MTC: Medullary thyroid cancer
mTOR: Mammalian target of rapamycin
MUGA: Multigated acquisition
NCI-CTCAE: National Cancer Institute-Common Terminology Criteria for Adverse Events
NETs: Neuroendocrine tumors
ORR: Overall response rate
OS: Overall survival
p: Pancreatic
PDGF: Platelet-derived growth factor
PET: Positron emission tomography
PFS: Progression-free survival
PI3K: Phosphoinositide 3 kinase
PS: Performance status
RCC: Renal cell carcinoma
RECIST: Response evaluation criteria in solid tumors
SAEs: Serious adverse events
SSAs: Somatostatin analogs
SSTRs: Somatostatin receptors
TKIs: Tyrosine kinase inhibitors
VEGF: Vascular Endothelial Growth Factor Receptor
WD: Well-differentiated
